# Clinical outcomes of DNA-damaging agents and DNA damage response inhibitors combinations in cancer: a data-driven review

**DOI:** 10.3389/fonc.2025.1577468

**Published:** 2025-06-10

**Authors:** Rick Fontenot, Neha Biyani, Kishor Bhatia, Reggie Ewesuedo, Marc Chamberlain, Panna Sharma

**Affiliations:** ^1^ Lantern Pharma Inc., Dallas, TX, United States; ^2^ Starlight Therapeutics, Plano, TX, United States

**Keywords:** DNA-damaging agents, DNA damage response inhibitors, PARP inhibitors, combination therapy, clinical trials, DNA repair pathways, cancer treatment, biomarkers

## Abstract

The combination of DNA-damaging agents (DDAs) and DNA damage response inhibitors (DDRis) has been extensively studied to improve therapeutic outcomes. While both groups of agents show promise individually, DDAs are limited by tumor resistance, and DDRis are limited by specific genetic context. Combining DDAs with DDRis may overcome these challenges and enhance patient outcomes. This review systematically analyzes clinical trials investigating the combination of DDAs and DDRis by dividing them into two sections: PARP and non-PARP inhibitors. An evaluation was conducted on 221 DDA-DDRi combination-arm trials involving 22 DDAs and 46 DDRis. DDAs were classified into eight subclasses, and DDRis into 14 distinct subclasses based on their mechanisms of action and specific targets, respectively. 89 of the 221 combination-arm trials had interpretable outcomes and were selected for further analysis. These were assigned outcome scores based on predefined criteria, reflecting their clinical effectiveness, safety, and benefit across different tumor types and patient populations. Our analysis emphasizes the patterns in treatment effectiveness, safety, and emerging trends across various cancer types and discusses the potential of biomarkers to guide treatment selection and improve patient outcomes. This review outlines an understanding of the recent state of DDA-DDRi combinations, offering critical insights for refining future cancer treatment strategies.

## 1 Introduction

DNA-damaging agents (DDAs), including chemotherapy and radiotherapy, have long been central to cancer treatment. They rely on their ability to induce irreparable genetic damage in rapidly dividing tumor cells (1). However, the efficacy of DDAs is frequently hampered by the activation of DNA damage response (DDR) mechanisms in cancer cells, which enable DNA repair and promote cell survival (2). This has spurred the development of DDR inhibitors (DDRis) designed to target these repair mechanisms, thereby enhancing the cytotoxic effects of DDAs (2, 4).

The DDR network is a complex, interconnected system with redundant pathways that provide compensatory and alternative repair mechanisms (5, 6). This redundancy presents therapeutic opportunities, exemplified by poly (ADP-ribose) polymerase inhibitors (PARPis), which exploit synthetic lethality to selectively kill cancer cells with defective DNA repair, as in cancers with BRCA mutations (3). PARPi approvals for treating ovarian, breast, and prostate cancers marked a significant advancement in personalized cancer therapy (7–11). However, the clinical utility of PARPis is confined mainly to specific genetic contexts, highlighting the need for broader treatment strategies (3). This need has driven the development of next-generation DDRis targeting diverse components of the DDR network.

Inhibitors of ATM, ATR, WEE1, and DNA-PK, for instance, disrupt distinct aspects of the DDR pathway, including cell cycle checkpoint regulation, DNA damage signaling, and repair processes (5, 12, 13). These agents offer potential therapeutic benefits across a broader range of tumor types, independent of specific genetic alterations like homologous recombination (HR) deficiencies, offering a more inclusive approach to overcoming resistance to DNA-damaging therapies (12). However, as monotherapies, DDRis often demonstrate limited efficacy due to rapid adaptation and developing resistance mechanisms in cancer cells (14).

The combination of DDRis and DDAs offers a compelling strategy to overcome these limitations. By simultaneously inducing DNA damage and inhibiting its repair, this approach can circumvent resistance mechanisms observed with monotherapy and expand the therapeutic potential beyond traditional DDA applications (2, 15). Numerous clinical trials are investigating these combination strategies across various cancer types and treatment regimens. The success of these combinations is influenced by factors such as tumor type, genetic profile, and the specific agents used. A critical challenge lies in identifying predictive biomarkers that can stratify patients based on their likelihood of response, enabling personalized treatment strategies and minimizing unnecessary toxicity (13, 16).

This review systematically analyzes the results of 221 DDAs-DDRis combination-arm clinical trials, encompassing 22 DDAs and 46 DDRis, without employing statistical methods. DDAs were grouped into eight subclasses according to their mechanisms of action, while DDRis were classified into 14 subclasses based on their specific targets. From the 221 initial combination-arm trials, 89 with interpretable outcomes were selected for in-depth analysis. These 89 trials were scored based on predefined criteria evaluating clinical effectiveness, safety, and benefit across diverse tumor types and patient populations, incorporating biomarker data where available. Given the prominent role of PARPis, the review is divided into PARP-focused and non-PARP-focused sections. By analyzing successful and challenging regimens, this work aims to provide a comprehensive overview of the field and inform future research on refining these combined therapies.

## 2 Methods

**Figure 1:**
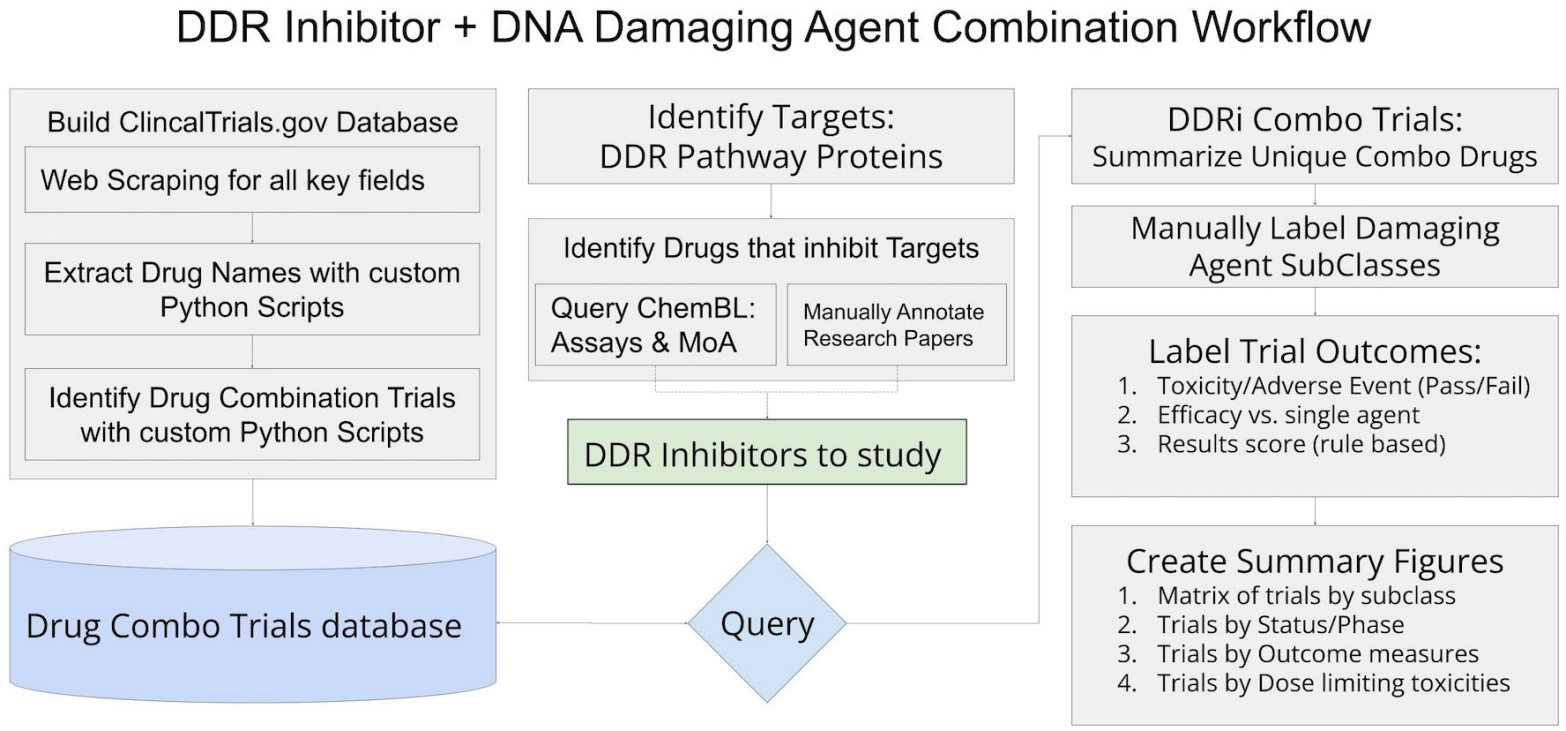
Workflow diagram of methods utilized to assemble data and results for this study.

The identification of relevant clinical trials and assembly of trial details and outcomes relied on accessing and organizing information from clinicaltrials.gov in conjunction with internally developed python scripts as well as steps of manual review and annotations to ensure details of each trial, drug, and results are reliable and accurate. **Figure 1** includes an overview of the workflow, and detailed descriptions of workflow sections follow.

### 2.1 Clinical trial data acquisition and processing

A queryable database of clinical trial information was needed to identify applicable trials and the relevant information associated with each trial. Pytrials (https://pytrials.readthedocs.io/en/latest/) provides a python query tool using the Clinicaltrials.gov API (https://clinicaltrials.gov/data-api/api); however, the API does not include relevant sections such as the trial’s detailed description, patient inclusion criteria, PMID references discussing trial results, and many more fields available on the clinicaltrials.gov page for each trial. Furthermore, the interventions returned by the API require further processing to properly extract and separate drug names.

In addition to the API clinicaltrials.gov allows users to download a JSON file including all fields for all trials. Data can be downloaded from this link: https://clinicaltrials.gov/search by clicking on the download button and selecting JSON with all available fields.

The nested trials inside the downloaded JSON are text rather than standardized dictionaries and do not all have the same fields or formats. A custom python script with additional processing was created to transform the JSON file into a standardized data table containing all fields available for each trial.

While the clincaltrials.gov page and JSON for each trial include a list of treatments in the “interventions” section, in many cases, it is not a complete list of drugs in the trial or synonyms the drug name is referenced to throughout the trial documents. Scripts using natural language processing and regular expressions tools were created to extract all drug names from the Interventions, ARM-Groups, and ARM-Interventions fields and compiled into a complete list for each trial in the newly created database.

The clinicaltrials.gov pages and downloads do not specifically include a field or label indicating whether the trial is a drug combination trial, so a rules-based script was created to flag which trials are drug combination trials. If a trial includes more than one drug, it is not necessarily a drug combination trial as the drugs may be administered as monotherapies for comparison in different arms of the trial. A rules-based script using natural language processing and regular expressions was created to flag trials with the words “combination” or “combined” used in either the trials title or brief summary and more than one unique drug in the trial drugs list created as described above. These flagged trials were included in a drug combinations specific view of the database for downstream querying and analysis. In total 490,490 clinical trials were processed, and 31,576 trials were identified as drug combination trials.

### 2.2 DNA damage repair inhibitor and DNA damaging agent identification

Identification of drugs that inhibit DDR pathways was accomplished by two methods, assay research and reviews of public conference presentations. A list of 120 proteins involved in the HR, NHEJ, alt-NHEJ, NER, MMR, BER, ICL, and TLS DNA damage repair pathways was compiled from literature (5, 17–31) to query the ChemBL database (https://www.ebi.ac.uk/chembl/). The query searched for inhibition assays for each protein in the compiled list and joined the drug names and drug name synonyms for each study with a significant percentage inhibition of the applicable protein and its associated repair pathway to retain the subclass of DNA damage repair inhibitors.

The list of 46 DDRis identified was used to query the drug combination clinical trials database view, resulting in 1,549 trials for initial review. A list of all unique drug names included in these trials resulted in 731 drugs that were manually annotated as DDA vs. other classes of drugs. Twenty-two DDAs across eight different DNA-damaging subclasses were identified as having at least one trial in combination with a DDRi. After filtering initially identified trials to the applicable drug class combinations, 221 trials with a DDRi and DDA in combination were identified for full review, with 89 of the trials being complete with at least one public source of the trial outcomes.

During the trial review phase, additional trials were removed as not relevant to this study if the DDA is only in a comparator arm while the DDRi drug was in a separate experimental arm rather than a test in combination.

In trials with multiple arms containing a DDRi + DDA combination, each arm was evaluated separately during reviews. This format allows for the analysis of counts based on specific drug combinations rather than a trial study ID.

### 2.3 Assigning numerical scores based on trial outcomes

Each applicable trial with results was manually reviewed to summarize outcomes from both outcome measures reported on clinicaltrials.gov tables as well as publicly available research papers summarizing results. For the purposes of visualization figures to graphically summarize which combinations of drug classes and specific drugs have demonstrated positive outcomes vs. negative or inconclusive outcomes, a numerical score was assigned to each trial. This numerical score is utilized to color code figures for a high-level representation of outcomes covering multiple trials as introduction prior to presenting details on specific individual trials or drug classes.

Initially three categories of numerical scores assigned are based on the following criteria during the manual annotation of outcomes process:

#### 2.3.1 Toxicity score

Trials that were discontinued due to significant adverse events or toxicities that prevented trial completion were graded as a negative outcome and assigned a numerical score of 1 representing the occurrence of discontinuation due to toxicity. Trials that were able to complete the study without trial limiting adverse events were graded as positive and assigned a score of 0, representing the lack of discontinuation. Although trials that received a score of 0 reported adverse events of varying severity, the current scoring system does not differentiate between the levels of severity of these adverse events, and no additional scoring was implemented to address this.

#### 2.3.2 Overall efficacy score

In trials where outcomes were measured as defined endpoints, the most used efficacy endpoints included partial response (PR), complete response (CR), objective response rate (ORR), disease control rate (DCR), median progression-free survival (mPFS), and overall survival (mOS), disease (SD), duration of response (DoR). Combination-trial arms achieving predefined efficacy endpoints were graded as positive outcome and assigned a numerical score of 1 (positive efficacy); those failing to meet endpoints were graded as negative and assigned a numerical score of 0 (lack of required efficacy). For trials lacking pre-defined endpoints but reporting efficacy outcomes, results were compared to standard-of-care expectations for the relevant indications and scored in the same manner as trials with defined endpoints. No reported outcomes: Completed combination-arm trial lacking any reported efficacy outcomes (e.g., some maximum tolerated dose [MTD] studies, which often focus on dose-limiting toxicity [DLT] and determining the recommended phase 2 dose [RP2D] rather than direct efficacy) were classified as having no available outcome data.

#### 2.3.3 Biomarker response score

In addition to the overall efficacy score, which is based on all trial participants, combination trial arms that reported differential efficacy outcomes for a subpopulation with specific biomarkers were also graded. Combination trial arms with a biomarker-defined patient subpopulation achieving the trials’ predefined efficacy endpoints or meeting standard-of-care expectations were graded as positive and assigned a score of 1. Combination trial arms where the biomarker-defined patient subpopulation did not exceed response rate of the overall trial participant group, or did not have outcomes reported for a biomarker patient subpopulation were graded as neutral and assigned a numerical score of 0.

#### 2.3.4 Outcome score

For use in summary visualizations and figures, these three individual categorical scores were then combined into an overall Outcome Score calculated as:


$$Outcome\;Score=Overall\;Efficacy\;Score+\;\frac{Biomarker\;response\;Score}{2}$$

Outcome Score values can be interpreted as:

**Score 0:** The combination-arm trial had a negative outcome where either the trial was discontinued due to adverse events or toxicities, or when the outcome was negative due to a lack of efficacy.
**Score 0.5:** The combination-arm trial was not discontinued due to adverse events or toxicities. While efficacy was not demonstrated for the overall participant group, there was a biomarker defined subpopulation that demonstrated efficacy.
**Score 1.0:** The combination-arm trial was not discontinued due to adverse events or toxicities and demonstrated efficacy for the studied participant group, but there were no outcomes reported for biomarker defined subgroups or the defined biomarker subgroup did not demonstrate efficacy above the other patients in the trial-arm.
**Score 1.5:** The combination-arm trial was not discontinued due to adverse events or toxicities and demonstrated efficacy for both the studied participant group, as well as an additional improvement in efficacy for a biomarker defined subgroup of participants.

## 3 Results

### 3.1 Clinical trial status of DDRis and DDAs: trends, development stages, and trial distribution

To assess the clinical landscape of DDAs-DDRis combinations, we analyzed clinical trials involving 22 unique DDAs in combination with 46 distinct DDRis. As a first step 22 DDAs were classified based on their mechanism of DNA damage into eight distinct DNA-damaging subclasses: alkylating agents, interstrand cross-linkers (ICLs), topoisomerase inhibitors, DNA intercalators, (dual-action agents) DNA intercalation & topoisomerase inhibition, ribonucleotide reductase inhibitors, G-quadruplex stabilizers, and multiple agents (**Table 1A**). Multiple agents denote a combination of multiple distinct therapeutic regimens, with at least one of these regimens including a DDA, with the possible addition of other agents like paclitaxel or pemetrexed. 46 DDRis were categorized into 14 subclasses based on their specific targets: ATR, AURK, CHK1/2, DNA-PK, PARP, PKMYT1, PLK, PLK+WEE1 (dual-targeting agents), PRMT5, RAD52, TP53, USP1, WEE1, and WRN (**Table 1B**).

**Table 1A:** List of DDAs and their Subclasses.

Damaging Drug Name	Damaging class
cyclophosphamide	alkylating agent
dacarbazine	alkylating agent
lurbinectedin	alkylating agent
temozolomide	alkylating agent
trabectedin	alkylating agent
mitomycin c	alkylating agent
doxorubicin	DNA intercalation
daunorubicin	DNA intercalation & topoisomerase inhibitor
epirubicin	DNA intercalation & topoisomerase inhibitor
idarubicin	DNA intercalation & topoisomerase inhibitor
mitoxantrone	DNA intercalation & topoisomerase inhibitor
cytarabine	DNA intercalation, topoisomerase inhibitor
pidnarulex	G-quadruplex stabilizer
carboplatin	Interstrand cross linker
cisplatin	Interstrand cross linker
oxaliplatin	Interstrand cross linker
gemcitabine	Ribonucleotide reductase inhibitor
hydroxyurea	Ribonucleotide reductase inhibitor
ep0057	topoisomerase inhibitor
etoposide	topoisomerase inhibitor
irinotecan	topoisomerase inhibitor
topotecan	topoisomerase inhibitor

**Table 1B:** List of DDRi Drugs, Their Subclasses, and affected DNA Damage Response Pathways.

Drug Name	DDRi Subclass	DNA damage response affected by DDRi Subclass
berzosertib	ATR	DNA damage checkpoint
elimusertib	ATR	DNA damage checkpoint
gartisertib	ATR	DNA damage checkpoint
sc0245	ATR	DNA damage checkpoint
tuvusertib	ATR	DNA damage checkpoint
alisertib	AURK	DNA damage checkpoint
chiauranib	AURK	DNA damage checkpoint
ilorasertib	AURK	DNA damage checkpoint
azd7762	CHK1/2	DNA damage checkpoint
prexasertib	CHK1/2	DNA damage checkpoint
rabusertib	CHK1/2	DNA damage checkpoint
sra737	CHK1/2	DNA damage checkpoint
azd7648	DNA-PK	DSBR
peposertib	DNA-PK	DSBR
samotolisib	DNA-PK	DSBR
vx-984	DNA-PK	DSBR
azd5305	PARP	SSBR
cep-9722	PARP	SSBR
e7016	PARP	SSBR
e7449	PARP	SSBR
fluzoparib	PARP	SSBR
nesuparib	PARP	SSBR
niraparib	PARP	SSBR
nms-03305293	PARP	SSBR
olaparib	PARP	SSBR
pamiparib	PARP	SSBR
rucaparib	PARP	SSBR
senaparib	PARP	SSBR
talazoparib	PARP	SSBR
veliparib	PARP	SSBR
venadaparib	PARP	SSBR
rp-6306	PKMYT1	DNA damage checkpoint
bal0891	PLK	DNA damage checkpoint
onvansertib	PLK	DNA damage checkpoint
rigosertib sodium	PLK	DNA damage checkpoint
adavosertib	PLK+WEE1	DNA damage checkpoint
volasertib	PLK+WEE1	DNA damage checkpoint
amg 193	PRMT5	DNA damage checkpoint
gossypol	RAD52	DSBR
idasanutlin	TP53	DNA damage checkpoint
navtemadlin	TP53	DNA damage checkpoint
siremadlin	TP53	DNA damage checkpoint
ro7623066	USP1	TLS and FA
azenosertib	WEE1	DNA damage checkpoint
debio 0123	WEE1	DNA damage checkpoint
hro761	WRN	DSBR and SSBR

SSBR, Single-Strand Break Repair, DSBR, Double-Strand Break Repair, TLS, Translesion Synthesis, FA, Fanconi Anemia.

Next, we analyzed clinical trials investigating combinations of these 22 DDAs and 46 DDRis. Each unique DDA-DDRi pairing within a trial was treated as an individual combination-arm trial. This means that if a single trial evaluated multiple treatment arms with different combinations of the DDA-DDRi, each arm was counted separately. The process yielded 221 combination-arm trials for analysis, listed in **Supplementary Tables S1**, **S2** (32–95), and **S3**.

**Figure 2:**
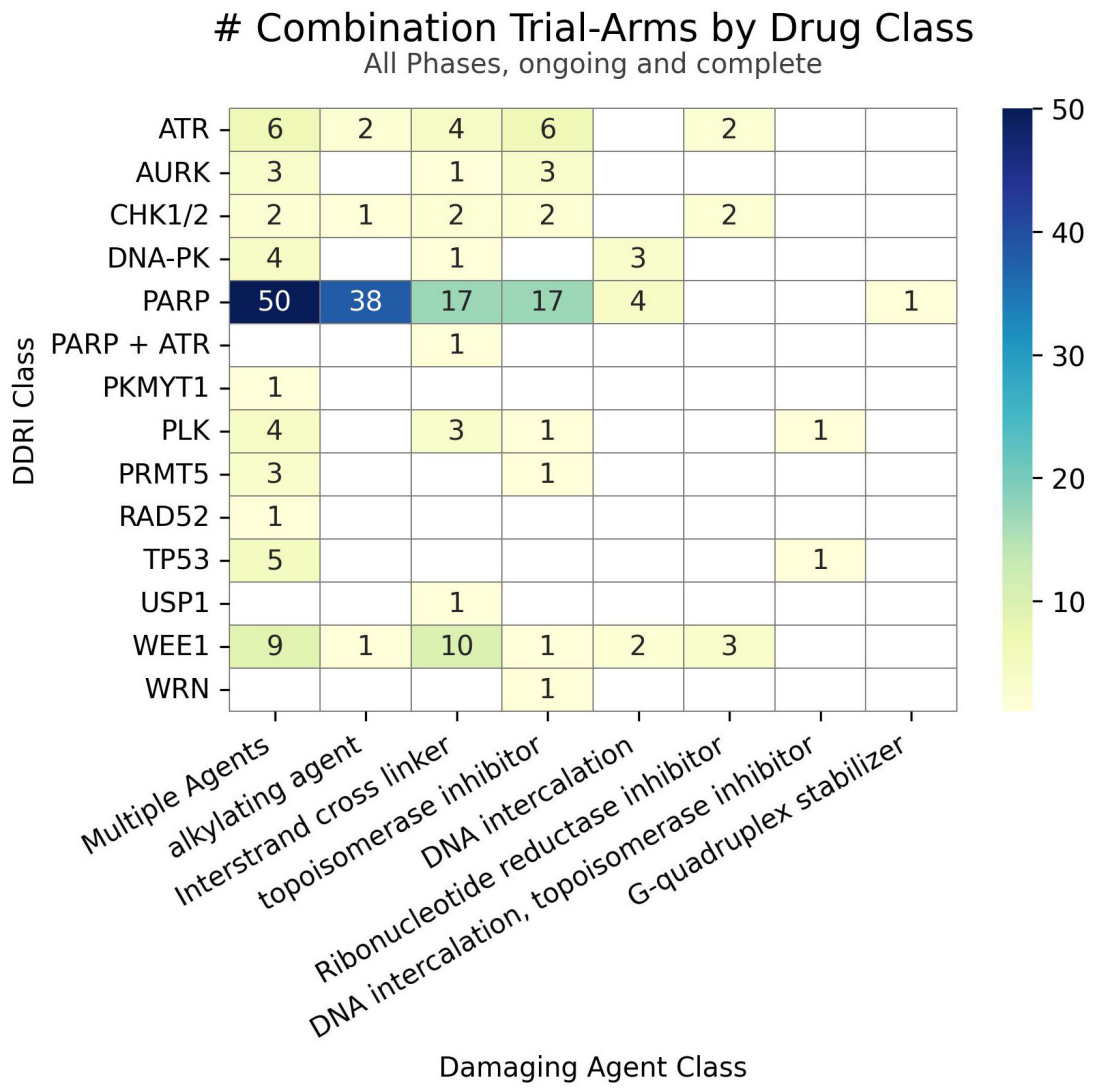
The number of combinations tested in trials for each class of DDRi (y-axis) versus DDA (x-axis). Unique drug combinations with multiple trials/phases are counted in the totals. Each drug combination is counted separately under the appropriate drug class totals for trials with multiple arms of interest.

**Figure 3:**
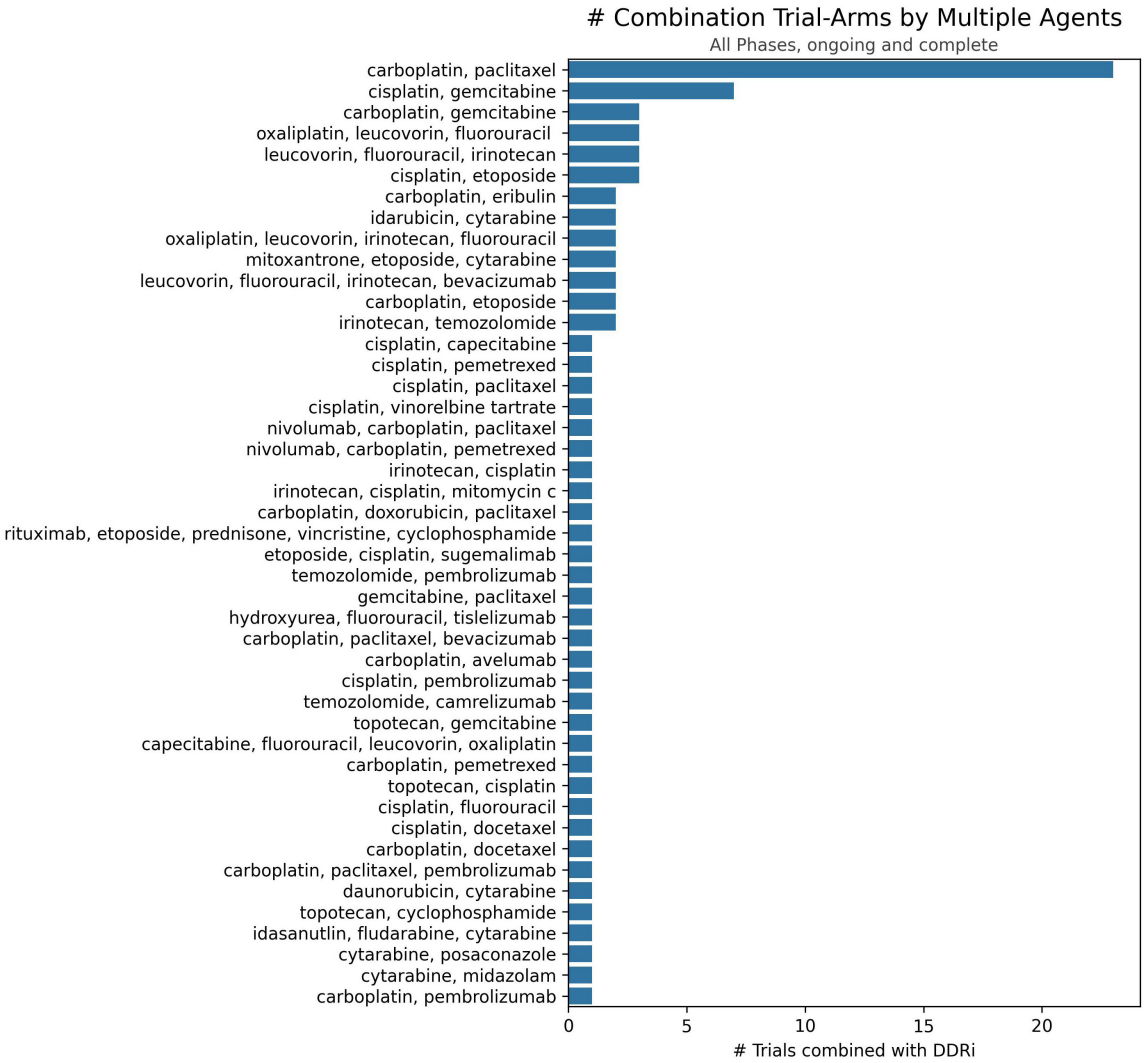
Number of trials investigating specific multiple-agent regimens in combination with DDRis. The x-axis represents the number of trials, and the y-axis lists the specific multiple-agent regimens.

Clinical trial data, seen in **Figure 2**, reveals a distinct trend in investigating DDAs-DDRis combinations by plotting the number of tested combinations across all trial phases and recruitment statuses wherein PARPis have been more extensively studied in combination with DDAs. Specifically, 127 combination arms have explored PARPi-DDA combinations, representing 57% of DDAs-DDRis combinations. At the same time, 94 trials have focused on non-PARP inhibitors (non-PARPis) and DDAs combinations, representing 43% of DDAs-DDRis combinations. Among DNA-damaging mechanisms investigated in DDRis combination-arm trials, multiple-agent regimens appeared the most frequently in 88 combination-arm trials. Among single-DDAs combinations with DDRis, alkylating agents were the most commonly investigated (42 combination-arm trials), followed by ICLs (40 combination-arm trials) and topoisomerase inhibitors (32 combination-arm trials). ICL agents, such as carboplatin, cisplatin, and oxaliplatin, are the most frequently utilized DDAs in multi-agent combination studies. The carboplatin and paclitaxel regimen (n=23) is the most commonly used DDAs-DDRis combination in multiple-agent combination arm trials, followed by the cisplatin and gemcitabine regimen (n=7), as shown in **Figure 3**.

**Figure 4:**
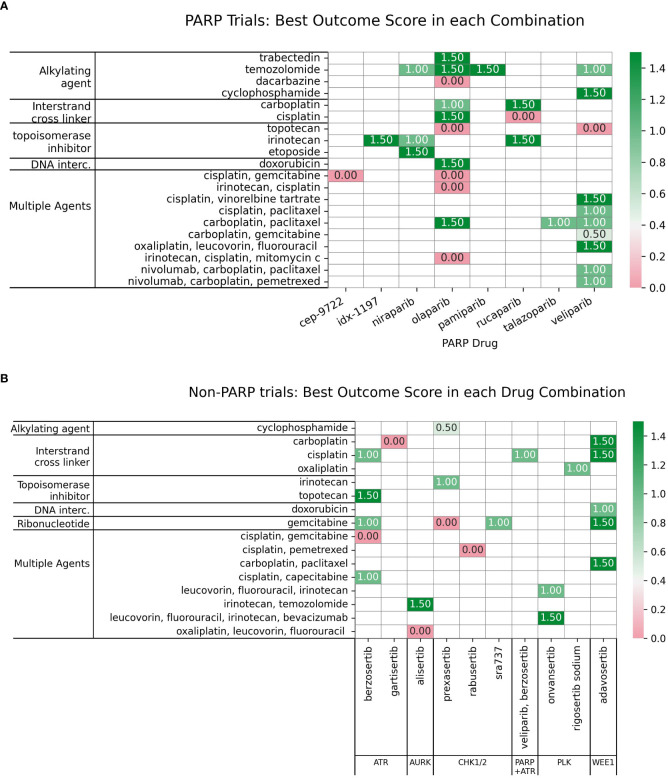
Number of trials distributed by clinical trial phase for each subclass of DDAs in combinations with **(A)** PARP inhibitors or **(B)** non-PARP DDR inhibitors. The x-axis represents the clinical trial phase, and the y-axis lists the number of combination-arm trials.

A detailed discussion about multiple agent combination-arm trials is beyond the scope of this article; however, essential information is provided in tables and relevant sections where applicable. Our analysis of the distribution of combination agents by clinical development stage within the PARPi and non-PARPi spaces revealed distinct trends as shown in **Figure 4**. A greater diversity of combination trials was observed in the PARPi space (**Figure 4A**). Specifically, among single DDA classes combined with PARPis, alkylating agents were the most frequently investigated in 38 combination-arm trials, followed by ICLs and topoisomerase inhibitors each in 17 combination-arm trials. Conversely, in the non-PARPi space (**Figure 3B**), ICLs (23 combination-arm trials) and topoisomerase inhibitors (15 combination-arm trials) were more extensively evaluated than alkylating agents (4 combination-arm trials). Alkylating agent combination arms represent 30% of PARPis combination-arm trials compared to 4% of non-PARPi combination arms. In contrast, ICLs were more frequently used in non-PARPi combination-arm trials (24%) than in PARPi combination-arm trials (13%). This indicates a distinct difference in combination strategies, where PARPi primarily combines with alkylating agents, whereas non-PARPi favors a combination with ICL agents.

**Figure 5:**
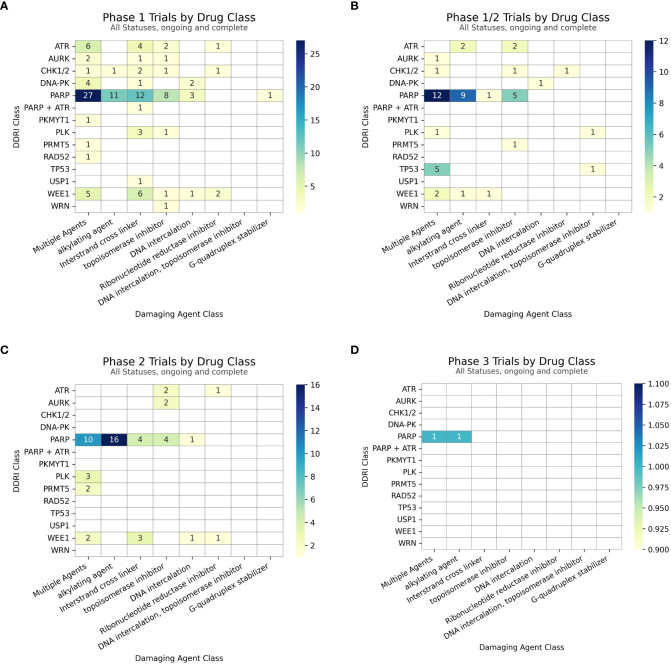
**(A)** Count of Phase 1 trial arms **(B)** Count of Phase 1/2 trial arms **(C)** Count of Phase 2 (inclusive of Phase 1/2) trial arms and **(D)** Count of Phase 3 trial arms with subtotals for combinations within each DDRi + DDA subclass.

221 DDAs-DDRis combination-arm clinical trials were distributed as follows: Phase 1 (117), phase 1/2 (49), phase 2 (52), with one in phase 2/3 and two in phase 3. PARPi combination-arm clinical trials were distributed as follows: Phase 1 (62), phase 1/2 (27), phase 2 (35), one in phase 2/3, and two in phase 3. Non-PARPi combination-arm clinical trials were predominantly distributed in Phase 1 (55), followed by phase 1/2 (22) and phase 2 (17), as shown in **Figures 5A–D**.

### 3.2 Clinical outcome scoring of selected DDAs-DDRis combination trials in PARPi and non-PARPi spaces

To assess the clinical outcomes of DDAs-DDRis combinations, 89 of the 221 identified combination-arm trials with interpretable outcomes were scored using a pre-defined scale (0, 0.5, 1, and 1.5; described in Methods) and listed in **Supplementary Table S1**. Zero scores indicate no efficacy or toxicity (failure); 0.5 indicates a positive response in a biomarker-selected population only; 1 indicates positive overall efficacy with no reported biomarker response; and 1.5 indicates both positive overall efficacy and a positive biomarker response. **Table 2** presents the score distribution across PARPi and non-PARPi spaces. A comparison of PARP and non-PARP inhibitor trials (n=57 and n=32, respectively) reveals distinct outcome distributions. PARP inhibitor trials showed a higher proportion of failures (35.1% scoring 0) compared to non-PARP inhibitor trials (28.1% scoring 0). Conversely, non-PARP inhibitor trials exhibited a higher proportion of positive efficacy without a reported biomarker response (40.6% scoring 1) compared to PARP inhibitor trials (28.1% scoring 1). The proportion of trials showing both positive efficacy and a biomarker response (score 1.5) was relatively similar between the two classes (26.3% for PARP inhibitors and 25.0% for non-PARP inhibitors). PARP inhibitors also demonstrated a higher percentage of trials with positive biomarker response only (10.5% scoring 0.5) compared to non-PARP inhibitors (6.2%).

**Table 2:** Distribution of scores across the PARP and non-PARP spaces.

DDRi Broad Class	Total number of trials	Number of trials by Outcome			
		Outcome Score			
		0	0.5	1.0	1.5
PARP Inhibitors	57	20	6	16	15
Non-PARP Inhibitors	32	9	2	13	8

The table summarizes the allocation of scores (0, 0.5, 1, and 1.5) for the outcomes of selected trials based on their classification within the PARP and non-PARP categories.

### 3.3 PARPis combinations: clinical trial outcomes with diverse DDAs

Of the initial 221 DDA-DDRi combination-arm trials, 127 in PARPi combination with DDAs and 57 had interpretable outcomes selected for further analysis and scored using pre-defined criteria (0, 0.5, 1, and 1.5, as described in methods). This analysis focused on eight PARPis, including five FDA approved drugs: olaparib (7), niraparib (8), rucaparib (9), talazoparib (10), and pamiparib (96) investigated in combination with DDAs (**Supplementary Table S1**, **Figure 5**). **Supplementary Table S1** provides key highlights of these trials, including specific regimens, trial phases, overall outcomes, adverse effects, and the score`s distribution.

**Figure 6:**
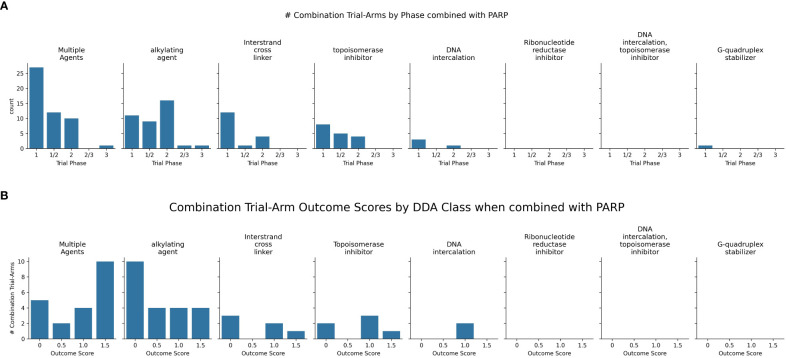
**(A)** Charts the highest combination trial-arm efficacy scores for PARPis combined with different DDA subclasses, illustrating which combinations have shown positive outcomes in at least one study. **(B)** Distribution of combination-arm trial’s outcome scores by DDA subclasses for PARPis in combinations. The x-axis represents the specific outcome score, and the y-axis lists the number of combination-arm trials.

Among the FDA approved PARPi inhibitors, veliparib and olaparib are the most widely studied in combinations with DDAs (**Figure 6A**).

Multiple agents, including carboplatin with paclitaxel, demonstrated positive outcomes when tested in combination with three PARPis-olaparib, talazoparib, and veliparib (**Figure 6A**). Among the seven multiple-agent regimens combined with veliparib (as shown in **Figure 6**), six (85%) showed overall positive outcomes. Although the remaining regimen was not positive in the overall cohort, it did show efficacy in a biomarker-defined subpopulation. In trials investigating 22 PARPi-alkylating agent combinations and shown in **Figure 6B**, 45.5% (10 trials) showed no efficacy/toxicity (score 0). The remaining trials were evenly distributed across positive outcomes: 18.2% (4 trials each) demonstrated a biomarker-specific response (score 0.5), overall efficacy without biomarker information (score 1), and both overall efficacy and a positive biomarker response (score 1.5). This mixed outcome profile highlights the challenges and variability in achieving both efficacy and biomarker responses. While alkylating agents, particularly temozolomide (TMZ), showed promise in uterine leiomyosarcoma (uLMS) (31) and relapsed small cell lung carcinoma (SCLC) (97), not all combinations were successful (e.g., veliparib/cyclophosphamide in TNBC (98), and veliparib/TMZ in hepatocellular carcinoma (99). Dose-limiting toxicities, including myelosuppression, were also observed (100). Biomarker-driven approaches, such as ERCC1 expression in metastatic melanoma (101) and an 8-gene signature in sarcomas CDKN2A, PIK3R1, SLFN11, ATM, APEX2, BLM, XRCC2, MAD2L2 that may help predict better outcomes (102, 103), offer potential for tailoring therapies.

**Figure 7:**
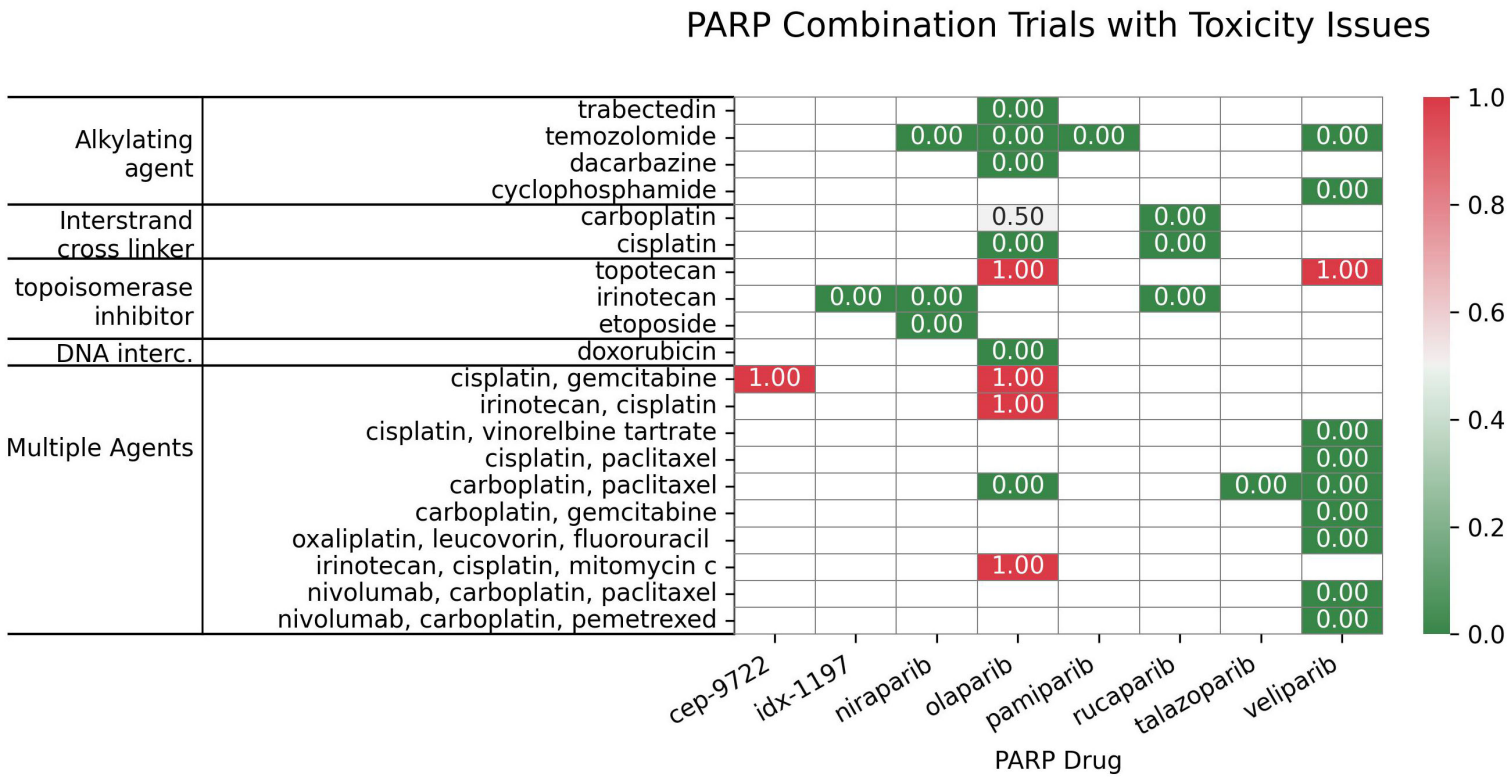
Distribution of the PARP inhibitors in combination with subclasses of DNA-damaging agents, categorized based on their toxicity scores.

For PARPi-ICL combinations (n=6), the outcome distribution was: 3 trials (50%) scored 0, indicating failure/no efficacy/toxicity; 1 trial (16.7%) scored 1, reflecting positive overall efficacy without a reported biomarker response; and 2 trials (33.3%) scored 1.5, indicating both positive efficacy and a positive biomarker response (**Figure 6B**). Combinations of PARPi with ICL agents, such as platinum compounds, demonstrate synergy (104, 105), particularly in BRCA-mutated tumors, but overlapping myelotoxicity remains a significant challenge (**Figure 7**).

In contrast, PARPi-topoisomerase inhibitor combinations (n=6) showed a different profile: 2 trials (33.3%) scored 0; 1 trial (16.7%) scored 1; and 3 trials (50%) scored 1.5. This suggests a trend towards positive efficacy and biomarker responses, although failures were also observed (**Figure 6B**) Notably, BRCA mutation status has emerged as a key predictor of improved outcomes with these combinations. PARPi combinations with topoisomerase inhibitors (e.g., irinotecan, etoposide) have yielded mixed results, showing promise in some indications like platinum-resistant ovarian (106) and HRD-positive gastric cancers, especially with specific genetic mutations (107); however, significant hematological toxicities (108, 109) have also limited the development of certain combinations.

These results indicate distinct outcome profiles for different PARPi-DDA combinations. In contrast, PARPi-ICL combinations in this small sample show a mix of responses; PARPi-topoisomerase inhibitor combinations trend toward more positive efficacy and biomarker responses. PARPi-alkylating agent combinations show a more balanced distribution of positive and negative outcomes.

### 3.4 Non-PARPis combinations: clinical trial outcomes with diverse DDAs

**Figure 8:**
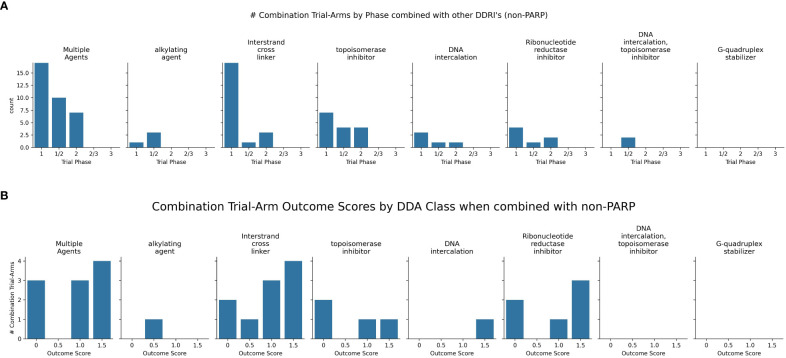
**(A)** Charts the highest combination trial-arm efficacy scores for non-PARPis combined with different DDA subclasses, illustrating which combinations have shown positive outcomes in at least one study. **(B)** Distribution of combination-arm trial’s outcome scores by DDA subclasses for non-PARPis. The x-axis represents the specific outcome score, and the y-axis lists the number of combination-arm trials.

Newer non-PARP DDRi targeting ATR, WEE1, and CHK1 also show promise in combination with DDAs (**Supplementary Table S2**, **Figure 8**).

As shown in **Figure 8B**, non-PARPi combinations were evaluated more extensively with ICLs (n=10) than with alkylating agents (n=1). The one trial investigating alkylating agents combined with non-PARPis scored 0.5 (100%), indicating a positive biomarker response only. Among the ten ICL-NonPARPi combinations, 80% (8 trials) showed some level of positive outcome (scores 0.5, 1, or 1.5), with 40% (4 trials) demonstrating positive overall efficacy without biomarker information and 30% (3 trials) demonstrating both positive efficacy and a positive biomarker response. 20% (2 trials) showed no efficacy (score 0). For the four topoisomerase inhibitor combinations with non-PARPis, the distribution was: 2 trials (50%) scored 0; 1 trial (25%) scored 1; and one trial (25%) scored 1.5. These results suggest that ICL-NonPARPi combinations demonstrate a more varied response, with a mix of failures and positive efficacy outcomes. Topoisomerase-NonPARPi combinations show a mixed outcome profile, with 50% of trials showing no efficacy and 50% showing some positive outcome (score 1 or 1.5). Clinically, berzosertib (ATR inhibitor) has shown promise with topotecan in relapsed neuroendocrine cancers (110) and also improving outcomes with gemcitabine in platinum-resistant HGSOC (111) and Non-Small Cell Lung Cancer (NSCLC) with high TMB/LOH (112). The same trial showed a negative outcome score when used in combination with gemcitabine + cisplatin, which did not yield an established RP2D due to toxicity concerns (113). As revealed in **Figure 8**, WEE1 inhibitor adavosertib consistently achieved a score of 1.5 across 3 combination -arm trials when combined with ICL-inducing agents, demonstrating a potent synergistic interaction and suggesting a promising synthetic lethal strategy. Adavosertib demonstrated benefit in TP53-mutated patients with platinum agents or gemcitabine (114); specifically achieving a 43% overall response rate in platinum-resistant or refractory epithelial ovarian cancer when combined with carboplatin (115). Further details on these trials, including specific outcomes, can be found in **Supplementary Table S2**. These findings highlight the potential of non-PARP DDRis, mainly when combined with platinum-based chemotherapy and emphasize the importance of identifying genetic vulnerabilities like TP53 mutations.

## 4 Discussion

This analysis of DDRi combinations with DDAs reveals distinct outcome profiles depending on the specific DDRi class (PARP vs. non-PARP) and the DDA employed. While this review aimed to provide a comprehensive overview using a defined scoring system (0 for failure/no efficacy/toxicity to 1.5 for positive efficacy and biomarker response, as detailed in the Results section and summarized in **Supplementary Tables S1**, **S2**, **Figures 6**–**8**), the dynamic nature of this field and the focus on interpretable outcomes means that it may not be fully exhaustive of all published studies. Future research will provide additional insights.

For PARPi combinations, the outcome distribution varied considerably across DDA subclasses. In 22 PARPi-alkylating agent combination trials, a substantial proportion (45.5%, 10 trials) showed no efficacy/toxicity (score 0), highlighting a key challenge with this combination strategy. The remaining trials exhibited a more balanced distribution across positive outcomes, with similar proportions demonstrating a biomarker-specific response (score 0.5), overall efficacy without biomarker information (score 1), and combined efficacy and biomarker response (score 1.5), each at 18.2% (4 trials). This heterogeneity underscores the influence of tumor biology and emphasizes the need for careful patient selection. While specific examples like olaparib/TMZ in uLMS (98) and SCLC (97) demonstrate promising efficacy, other combinations and tumor types did not show similar benefits, and dose-limiting toxicities were observed. This highlights the importance of biomarker-driven approaches, as exemplified by studies using ERCC1 expression (101) and 8-gene signatures (102, 103), to personalize treatment strategies.

In contrast, the limited data for PARPi-ICL combinations (n=6) revealed a distinct profile: (50%) showed no efficacy/toxicity (score 0), while 3 trials showed other positive outcome scores. This small sample size prevents definitive conclusions; however, it suggests that while synergy with platinum agents is theoretically sound (especially in BRCA-mutated tumors), clinical outcomes are not uniformly positive, and overlapping myelotoxicity remains a critical challenge. PARPi-topoisomerase inhibitor combinations (n=6) indicated a more promising trend, with a higher proportion of trials showing both positive efficacy and biomarker responses (50%, score 1.5), although failures were also observed (33.3%, score 0). This suggests that this combination strategy may be particularly promising in certain contexts, particularly in HRD-positive tumors. Furthermore, ongoing investigation of next-generation PARP1-selective inhibitors, e.g., NMS-03305293 (116) and AZD5305 (117), in combination with DDAs, aims to address toxicity and improve the therapeutic index.

Optimizing the delivery and tolerability of DNA-damaging agents can be a critical parallel strategy to enhancing their efficacy in combination with DDR inhibitors. Liposomal doxorubicin, for example, offers a more favorable pharmacokinetic profile and reduced cardiotoxicity, expanding its therapeutic window and making it a more suitable partner in regimens where cumulative cardiac risk is a limiting factor (118, 119). These advancements in formulation can help address the challenges of maximizing the therapeutic index of DNA-damaging agents for successful combination strategies with DDRis. In our analysis, all identified trials using doxorubicin in combination with DDRi employed a liposomal or pegylated liposomal formulation. Notably, the two PARP inhibitor trials—NCT03161132 (120, 121) and NCT00819221 (122)—demonstrated strong performance, receiving maximum scores of 1.5 for overall efficacy and biomarker relevance. Conventional doxorubicin was not studied in combination with DDRis. Nonetheless, these observations highlight the promise of novel formulation strategies to improve tolerability and expand the therapeutic potential of DDR-based combination therapies.

Non-PARPi combinations exhibited a different pattern. They were more extensively evaluated with ICLs (n=10) than alkylating agents (n=1), possibly reflecting a strategic focus on exploiting platinum-induced DNA damage. These ICL-NonPARPi combinations demonstrated promising activity, with the majority of trials (80%, 8 trials) showing some level of positive outcome. The distribution of these positive outcomes—40% (4 trials) demonstrating overall efficacy without biomarker information (score 1) and 30% (3 trials) demonstrating both efficacy and a positive biomarker response (score 1.5)—highlights the need for further investigation to understand the factors contributing to varied responses and to develop strategies for patient selection. The single trial evaluating alkylating-NonPARPi combinations prevents any meaningful conclusions. Topoisomerase-NonPARPi combinations (n=4) showed a mixed outcome profile, with 50% of trials showing no efficacy and 50% showing some positive outcome (score 1 or 1.5).

Comparing PARPi and non-PARP DDRi combinations, it is evident that different DDAs elicit distinct responses. While PARPi combinations show a more balanced distribution of outcomes across DDA subclasses (with the exception of the small ICL dataset), non-PARPi combinations appear to be more focused on ICLs, with a more varied range of responses. This highlights the importance of considering the specific DDR pathway targeted by the inhibitor and the type of DNA damage induced by the DDA when designing combination strategies. As the field evolves, refining these strategies and identifying new targets within the DDR network and combination agents is crucial. Advancing promising DDRi–DDA combinations will require further validation through large-scale clinical trials in well-defined patient populations, supported by the development of robust predictive biomarkers. Optimizing treatment sequencing and dosing will also be key to maximizing clinical benefits (2). Preclinical studies should continue elucidating synergistic mechanisms in diverse cancer models and investigating resistance mechanisms. Future research should focus on the rational selection of DDRi-DDA combinations based on tumor-specific DDR defects and explore multi-DDR targeting strategies to achieve deeper and more durable responses (123, 124). A data-driven approach with a higher level of automation could be highly beneficial for scientists and clinicians in determining and designing optimal combination trials.

## Data Availability

The original contributions presented in the study are included in the article/**Supplementary Material**. Further inquiries can be directed to the corresponding author.

## Supplementary material

Supplementary Table 1 This table summarizes DDAs- PARPi combination-arm trials, including study ID, drugs, cancer types, phase, status, efficacy, biomarkers, toxicity-related discontinuations, adverse effects, treatment regimens, and trial dates/enrollment and scores, as defined in the method section. Abbreviations: partial response (PR), dose-limiting toxicity (DLT), recommended phase 2 dose (RP2D), stable disease (SD), objective response rate (ORR), disease control rate (DCR), median progression-free survival (mPFS), overall survival (mOS), adverse events (AEs), complete response (CR), duration of response (DoR), twice daily (BID), maximum tolerated dose (MTD), pharmacokinetics (PK), small-cell lung cancer (SCLC), triple-negative breast cancer (TNBC), non-small-cell lung cancer (NSCLC), glioblastoma (GBM), and pancreatic ductal adenocarcinoma (PDAC).

Supplementary Table 2 This table summarizes DDAs-NonPARPi combination-arm trials, including study ID, drugs, cancer types, phase, status, efficacy, biomarkers, toxicity-related discontinuations, adverse effects, treatment regimens, and trial dates/enrollment and scores, as defined in the method section. Abbreviations: partial response (PR), dose-limiting toxicity (DLT), recommended phase 2 dose (RP2D), stable disease (SD), objective response rate (ORR), disease control rate (DCR), median progression-free survival (mPFS), overall survival (mOS), adverse events (AEs), complete response (CR), duration of response (DoR), twice daily (BID), maximum tolerated dose (MTD), pharmacokinetics (PK), small-cell lung cancer (SCLC), non-small-cell lung cancer (NSCLC), primary platinum-resistant ovarian cancer (PROC), extrapulmonary small cell neuroendocrine carcinoma (EP-SCNC).

Supplementary Table 3 A list of combination-arm clinical trials without outcomes.

## References

[1] Larsen BD Benada J Yung PYK Bell RAV Pappas G Urban V. Cancer cells use self-inflicted DNA breaks to evade growth limits imposed by genotoxic stress. Science. (2022) 376:476–83. doi: 10.1126/science.abi6378 35482866

[2] O’Connor MJ. Targeting the DNA damage response in cancer. Mol Cell. (2015) 60:547–60. doi: 10.1016/j.molcel.2015.10.040 26590714

[3] Lord CJ Ashworth A. PARP inhibitors: Synthetic lethality in the clinic. Science. (2017) 355:1152–8. doi: 10.1126/science.aam7344 PMC6175050 28302823

[4] Goldstein M Kastan MB. The DNA damage response: implications for tumor responses to radiation and chemotherapy. Annu Rev Med. (2015) 66:1–15. doi: 10.1146/annurev-med-081313-121208 25423595

[5] Li Q Qian W Zhang Y Hu L Chen S Xia Y. A new wave of innovations within the DNA damage response. Signal Transduct Target Ther. (2023) 8:338. doi: 10.1038/s41392-023-01548-8 37679326 PMC10485079

[6] Pearl LH Schierz AC Ward SE Al-Lazikani B Pearl FMG. Therapeutic opportunities within the DNA damage response. Nat Rev Cancer. (2015) 15:166–80. doi: 10.1038/nrc3891 25709118

[7] Deeks ED. Olaparib: first global approval. Drugs. (2015) 75:231–40. doi: 10.1007/s40265-015-0345-6 25616434

[8] Scott LJ. Niraparib: first global approval. Drugs. (2017) 77:1029–34. doi: 10.1007/s40265-017-0752-y 28474297

[9] Syed YY. Rucaparib: first global approval. Drugs. (2017) 77:585–92. doi: 10.1007/s40265-017-0716-2 28247266

[10] Hoy SM. Talazoparib: first global approval. Drugs. (2018) 78:1939–46. doi: 10.1007/s40265-018-1026-z 30506138

[11] Lee A. Fuzuloparib: first approval. Drugs. (2021) 81:1221–6. doi: 10.1007/s40265-021-01541-x PMC8380563 34118019

[12] Pilié PG Tang C Mills GB Yap TA. State-of-the-art strategies for targeting the DNA damage response in cancer. Nat Rev Clin Oncol. (2019) 16:81–104. doi: 10.1038/s41571-018-0114-z 30356138 PMC8327299

[13] Cleary JM Aguirre AJ Shapiro GI D’Andrea AD. Biomarker-guided development of DNA repair inhibitors. Mol Cell. (2020) 78:1070–85. doi: 10.1016/j.molcel.2020.04.035 PMC7316088 32459988

[14] Li H Liu Z-Y Wu N Chen Y-C Cheng Q Wang J. PARP inhibitor resistance: the underlying mechanisms and clinical implications. Mol Cancer. (2020) 19:107. doi: 10.1186/s12943-020-01227-0 32563252 PMC7305609

[15] Helleday T Petermann E Lundin C Hodgson B Sharma RA. DNA repair pathways as targets for cancer therapy. Nat Rev Cancer. (2008) 8:193–204. doi: 10.1038/nrc2342 18256616

[16] Smith AD Roda D Yap TA. Strategies for modern biomarker and drug development in oncology. J Hematol Oncol. (2014) 7:70. doi: 10.1186/s13045-014-0070-8 25277503 PMC4189730

[17] Helleday T Lo J van Gent DC Engelward BP. DNA double-strand break repair: From mechanistic understanding to cancer treatment. DNA Repair. (2007) 6:923–35. doi: 10.1016/j.dnarep.2007.02.006 17363343

[18] Lieber MR Ma Y Pannicke U Schwarz K. Mechanism and regulation of human non-homologous DNA end-joining. Nat Rev Mol Cell Biol. (2003) 4:712–20. doi: 10.1038/nrm1202 14506474

[19] McVey M Lee SE. MMEJ repair of double-strand breaks (director’s cut): deleted sequences and alternative endings. Trends Genet. (2008) 24:529–38. doi: 10.1016/j.tig.2008.08.007 PMC5303623 18809224

[20] Krokan H Standal R Slupphaug G. DNA glycosylases in the base excision repair of DNA. Biochem J. (1997) 325:1–16. doi: 10.1042/bj3250001 9224623 PMC1218522

[21] Shilkin ES Boldinova EO Stolyarenko AD Goncharova RI Chuprov-Netochin RN Smal MP. Translesion DNA synthesis and reinitiation of DNA synthesis in chemotherapy resistance. Biochem (Mosc). (2020) 85:869–82. doi: 10.1134/s0006297920080039 33045948

[22] Li G-M. Mechanisms and functions of DNA mismatch repair. Cell Res. (2008) 18:85–98. doi: 10.1038/cr.2007.115 18157157

[23] Räschle M Knipscheer P Knipsheer P Enoiu M Angelov T Sun J. Mechanism of replication-coupled DNA interstrand crosslink repair. Cell. (2008) 134:969–80. doi: 10.1016/j.cell.2008.08.030 PMC2748255 18805090

[24] Sale JE Lehmann AR Woodgate R. Y-family DNA polymerases and their role in tolerance of cellular DNA damage. Nat Rev Mol Cell Biol. (2012) 13:141–52. doi: 10.1038/nrm3289 PMC3630503 22358330

[25] Wang M Chen S Ao D. Targeting DNA repair pathway in cancer: Mechanisms and clinical application. MedComm. (2021) 2:654–91. doi: 10.1002/mco2.103 PMC8706759 34977872

[26] Caracciolo D Riillo C Martino MTD Tagliaferri P Tassone P. Alternative non-homologous end-joining: error-prone DNA repair as cancer’s Achilles’ Heel. Cancers. (2021) 13:1392. doi: 10.3390/cancers13061392 33808562 PMC8003480

[27] Hashimoto S Anai H Hanada K. Mechanisms of interstrand DNA crosslink repair and human disorders. Genes Environ. (2016) 38:9. doi: 10.1186/s41021-016-0037-9 27350828 PMC4918140

[28] Lieber MR. The mechanism of human nonhomologous DNA end joining*. J Biol Chem. (2008) 283:1–5. doi: 10.1074/jbc.r700039200 17999957

[29] Kusakabe M Onishi Y Tada H Kurihara F Kusao K Furukawa M. Mechanism and regulation of DNA damage recognition in nucleotide excision repair. Genes Environ. (2019) 41:2. doi: 10.1186/s41021-019-0119-6 30700997 PMC6346561

[30] Harrigan JA Wilson DM Prasad R Opresko PL Beck G May A. The Werner syndrome protein operates in base excision repair and cooperates with DNA polymerase β. Nucleic Acids Res. (2006) 34:745–54. doi: 10.1093/nar/gkj475 PMC1356534 16449207

[31] Sallmyr A Rassool FV. Up-regulated WRN and DNA ligase IIIα Are involved in alternative NHEJ repair pathway of DNA double strand breaks (DSB) in chronic myeloid leukemia (CML). Blood. (2007) 110:1016. doi: 10.1182/blood.v110.11.1016.1016

[32] Kummar S Wade JL Oza AM Sullivan D Chen AP Gandara DR. Randomized phase II trial of cyclophosphamide and the oral poly (ADP-ribose) polymerase inhibitor veliparib in patients with recurrent, advanced triple-negative breast cancer. Invest N Drugs. (2016) 34:355–63. doi: 10.1007/s10637-016-0335-x PMC4860030 26996385

[33] Khan OA Gore M Lorigan P Stone J Greystoke A Burke W. A phase I study of the safety and tolerability of olaparib (AZD2281, KU0059436) and dacarbazine in patients with advanced solid tumours. Br J Cancer. (2011) 104:750–5. doi: 10.1038/bjc.2011.8 PMC3048218 21326243

[34] Foster JC Freidlin B Kunos CA Korn EL. Single-arm phase II trials of combination therapies: A review of the CTEP experience 2008–2017. JNCI: J Natl Cancer Inst. (2020) 112:128–35. doi: 10.1093/jnci/djz193 PMC7019095 31545373

[35] Study Details | Veliparib and Temozolomide in Treating Patients With Recurrent Glioblastoma. ClinicalTrials.gov. Available at: https://clinicaltrials.gov/study/NCT01026493?term=NCT01026493&rank=1.

[36] Cecchini M Zhang JY Wei W Sklar J Lacy J Zhong M. Quantitative DNA repair biomarkers and immune profiling for temozolomide and olaparib in metastatic colorectal cancer. Cancer Res Commun. (2023) 3:1132–9. doi: 10.1158/2767-9764.crc-23-0045 PMC10305782 37387791

[37] Su JM Thompson PA Adesina A Li X-N Kilburn LB Onar-Thomas A. A phase I clinical trial of veliparib and temozolomide in children with recurrent central nervous system tumors: A Pediatric Brain Tumor Consortium report. J Clin Oncol. (2013) 31:2036–6. doi: 10.1200/jco.2013.31.15_suppl.2036

[38] Stradella A Johnson M Goel S Park H Lakhani N Arkenau H. Phase 1b study to assess the safety, tolerability, and clinical activity of pamiparib in combination with temozolomide in patients with locally advanced or metastatic solid tumors. Cancer Med. (2024) 13:e7385. doi: 10.1002/cam4.7385 38970256 PMC11226541

[39] Hussain M Carducci MA Slovin S Cetnar J Qian J McKeegan EM. Targeting DNA repair with combination veliparib (ABT-888) and temozolomide in patients with metastatic castration-resistant prostate cancer. Invest N Drugs. (2014) 32:904–12. doi: 10.1007/s10637-014-0099-0 PMC4659356 24764124

[40] Kummar S Ji J Morgan R Lenz H-J Puhalla SL Belani CP. A phase I study of veliparib in combination with metronomic cyclophosphamide in adults with refractory solid tumors and lymphomas. Clin Cancer Res. (2012) 18:1726–34. doi: 10.1158/1078-0432.ccr-11-2821 PMC3306481 22307137

[41] Pishvaian MJ Slack RS Jiang W He AR Hwang JJ Hankin A. A phase 2 study of the PARP inhibitor veliparib plus temozolomide in patients with heavily pretreated metastatic colorectal cancer. Cancer. (2018) 124:2337–46. doi: 10.1002/cncr.31309 PMC5992024 29579325

[42] Xu J Keenan TE Overmoyer B Tung NM Gelman RS Habin K. Phase II trial of veliparib and temozolomide in metastatic breast cancer patients with and without BRCA1/2 mutations. Breast Cancer Res Treat. (2021) 189:641–51. doi: 10.1007/s10549-021-06292-7 34417675

[43] Halford SER Cruickshank G Dunn L Erridge S Godfrey L Herbert C. Results of the OPARATIC trial: A phase I dose escalation study of olaparib in combination with temozolomide (TMZ) in patients with relapsed glioblastoma (GBM). J Clin Oncol. (2017) 35:2022–2. doi: 10.1200/jco.2017.35.15_suppl.2022

[44] Piotrowski A Puduvalli V Wen P Campian J Colman H Pearlman M. Actr-39. Pamiparib in combination with radiation therapy (rt) and/or temozolomide (tmz) in patients with newly diagnosed or recurrent/refractory (r/r) glioblastoma (gbm); phase 1b/2 study update. Neuro-Oncol. (2019) 21:vi21–2. doi: 10.1093/neuonc/noz175.081

[45] Chugh R Ballman KV Helman LJ Patel S Whelan JS Widemann B. SARC025 arms 1 and 2: A phase 1 study of the poly(ADP-ribose) polymerase inhibitor niraparib with temozolomide or irinotecan in patients with advanced Ewing sarcoma. Cancer. (2021) 127:1301–10. doi: 10.1002/cncr.33349 PMC8246769 33289920

[46] Kurzrock R Galanis E Johnson DR Kansra V Wilcoxen K Mcclure T. A phase I study of niraparib in combination with temozolomide (TMZ) in patients with advanced cancer. J Clin Oncol. (2014) 32:2092–2. doi: 10.1200/jco.2014.32.15_suppl.2092

[47] Heilig CE Teleanu M Bhatti IA Richter S Siveke JT Wagner S. Randomized phase II study of trabectedin/olaparib compared to physician’s choice in subjects with previously treated advanced or recurrent solid tumors harboring DNA repair deficiencies (2022). Available online at: https://oncologypro.esmo.org/meeting-resources/esmo-congress-2022/randomized-phase-ii-study-of-trabectedin-olaparib-compared-to-physician-s-choice-in-subjects-with-previously-treated-advanced-or-recurrent-solid-tu (Accessed January 23, 2025).

[48] van der Noll R Jager A Ang JE Marchetti S Mergui-Roelvink MWJ Lolkema MP. Phase I study of continuous olaparib capsule dosing in combination with carboplatin and/or paclitaxel (Part 1). Invest N Drugs. (2020) 38:1117–28. doi: 10.1007/s10637-019-00856-7 31667659

[49] Forster M. ORCA-2: A phase I study of olaparib in addition to cisplatin-based concurrent chemoradiotherapy for patients with high risk locally advanced (LA) squamous cell carcinoma of the head and neck (HNSCC) (2021). Available online at: https://cslide.ctimeetingtech.com/esmo2021/attendee/confcal_4/presentation/list?q=866P (Accessed January 23, 2025).

[50] Lee J-M Hays JL Chiou VL Annunziata CM Swisher EM Harrell MI. Phase I/Ib study of olaparib and carboplatin in women with triple negative breast cancer. Oncotarget. (2017) 8:79175–87. doi: 10.18632/oncotarget.16577 PMC5668030 29108297

[51] Miller K Tong Y Jones DR Walsh T Danso MA Ma CX. Cisplatin with or without rucaparib after preoperative chemotherapy in patients with triple negative breast cancer: Final efficacy results of Hoosier Oncology Group BRE09-146. J Clin Oncol. (2015) 33:1082–2. doi: 10.1200/jco.2015.33.15_suppl.1082

[52] Wilson RH Evans TJ Middleton MR Molife LR Spicer J Dieras V. A phase I study of intravenous and oral rucaparib in combination with chemotherapy in patients with advanced solid tumours. Br J Cancer. (2017) 116:884–92. doi: 10.1038/bjc.2017.36 PMC5379148 28222073

[53] Balmaña J Tung NM Isakoff SJ Graña B Ryan PD Saura C. Phase I trial of olaparib in combination with cisplatin for the treatment of patients with advanced breast, ovarian and other solid tumors. Ann Oncol. (2014) 25:1656–63. doi: 10.1093/annonc/mdu187 24827126

[54] Awada A Campone M Varga A Aftimos P Frenel J-S Bahleda R. An open-label, dose-escalation study to evaluate the safety and pharmacokinetics of CEP-9722 (a PARP-1 and PARP-2 inhibitor) in combination with gemcitabine and cisplatin in patients with advanced solid tumors. Anti-Cancer Drugs. (2016) 27:342–8. doi: 10.1097/cad.0000000000000336 26796987

[55] Giaccone G Rajan A Kelly RJ Gutierrez M Kummar S Yancey M. A phase I combination study of olaparib (AZD2281; KU-0059436) and cisplatin (C) plus gemcitabine (G) in adults with solid tumors. J Clin Oncol. (2010) 28:3027–7. doi: 10.1200/jco.2010.28.15_suppl.3027

[56] Stodtmann S Eckert D Joshi R Nuthalapati S Ratajczak CK Menon R. Exposure-response model with time-varying predictors to estimate the effects of veliparib in combination with carboplatin/paclitaxel and as monotherapy: Veliparib phase 3 study in BRCA-mutated advanced breast cancer (BROCADE3) trial. J Clin Pharmacol. (2022) 62:1236–46. doi: 10.1002/jcph.2061 35403245

[57] Yarchoan M Myzak MC Johnson BA Jesus-Acosta AD Le DT Jaffee EM. Olaparib in combination with irinotecan, cisplatin, and mitomycin C in patients with advanced pancreatic cancer. Oncotarget. (2017) 8:44073–81. doi: 10.18632/oncotarget.17237 PMC5546463 28454122

[58] Mego M Svetlovska D Reckova M Kalavska K Obertova J Palacka P. Phase II study of gemcitabine, carboplatin and veliparib in multiple relapsed/refractory germ cell tumors (GCTs). J Clin Oncol. (2021) 39:e17009. doi: 10.1200/jco.2021.39.15_suppl.e17009 34052929

[59] Clarke JM Patel JD Robert F Kio EA Thara E Camidge DR. Veliparib and nivolumab in combination with platinum doublet chemotherapy in patients with metastatic or advanced non-small cell lung cancer: A phase 1 dose escalation study. Lung Cancer. (2021) 161:180–8. doi: 10.1016/j.lungcan.2021.09.004 34607210

[60] Gray HJ Bell-McGuinn K Fleming GF Cristea M Xiong H Sullivan D. Phase I combination study of the PARP inhibitor veliparib plus carboplatin and gemcitabine in patients with advanced ovarian cancer and other solid Malignancies. Gynecol Oncol. (2018) 148:507–14. doi: 10.1016/j.ygyno.2017.12.029 29352572

[61] Malhotra MK Pahuja S Kiesel BF Appleman LJ Ding F Lin Y. A phase 1 study of veliparib (ABT-888) plus weekly carboplatin and paclitaxel in advanced solid Malignancies, with an expansion cohort in triple negative breast cancer (TNBC) (ETCTN 8620). Breast Cancer Res Treat. (2023) 198:487–98. doi: 10.1007/s10549-023-06889-0 PMC10710035 36853577

[62] Turk AA Leal T Chan N Wesolowski R Spencer KR Malhotra J. NCI9782: A phase 1 study of talazoparib in combination with carboplatin and paclitaxel in patients with advanced solid tumors. J Clin Oncol. (2019) 37:e14640. doi: 10.1200/jco.2019.37.15_suppl.e14640

[63] Han HS Diéras V Robson M Palácová M Marcom PK Jager A. Veliparib with temozolomide or carboplatin/paclitaxel versus placebo with carboplatin/paclitaxel in patients with BRCA1/2 locally recurrent/metastatic breast cancer: randomized phase II study. Ann Oncol. (2018) 29:154–61. doi: 10.1093/annonc/mdx505 PMC5834075 29045554

[64] Mizugaki H Yamamoto N Nokihara H Fujiwara Y Horinouchi H Kanda S. A phase 1 study evaluating the pharmacokinetics and preliminary efficacy of veliparib (ABT-888) in combination with carboplatin/paclitaxel in Japanese subjects with non-small cell lung cancer (NSCLC). Cancer Chemother Pharmacol. (2015) 76:1063–72. doi: 10.1007/s00280-015-2876-7 PMC4612330 26433581

[65] Oza AM Cibula D Benzaquen AO Poole C Mathijssen RHJ Sonke GS. Olaparib combined with chemotherapy for recurrent platinum-sensitive ovarian cancer: a randomised phase 2 trial. Lancet Oncol. (2015) 16:87–97. doi: 10.1016/s1470-2045(14)71135-0 25481791

[66] Ramalingam SS Blais N Mazieres J Reck M Jones CM Juhasz E. Randomized, placebo-controlled, phase II study of veliparib in combination with carboplatin and paclitaxel for advanced/metastatic non–small cell lung cancer. Clin Cancer Res. (2017) 23:1937–44. doi: 10.1158/1078-0432.ccr-15-3069 27803064

[67] Nishio S Takekuma M Takeuchi S Kawano K Tsuda N Tasaki K. Phase 1 study of veliparib with carboplatin and weekly paclitaxel in Japanese patients with newly diagnosed ovarian cancer. Cancer Sci. (2017) 108:2213–20. doi: 10.1111/cas.13381 PMC5665762 28837250

[68] Rivkin SE Moon J Iriarte DS Bailey E Sloan HL Goodman GE. Phase Ib with expansion study of olaparib plus weekly (Metronomic) carboplatin and paclitaxel in relapsed ovarian cancer patients. Int J Gynecol Cancer. (2019) 29:325–33. doi: 10.1136/ijgc-2018-000035 30700568

[69] Pishvaian MJ Wang H Parenti S He AR Hwang JJ Ley L. Final report of a phase I/II study of veliparib (Vel) in combination with 5-FU and oxaliplatin (FOLFOX) in patients (pts) with metastatic pancreatic cancer (mPDAC). J Clin Oncol. (2019) 37:4015–5. doi: 10.1200/jco.2019.37.15_suppl.4015

[70] Jelinek MJ Foster NR Zoroufy AJ Souza JAD Schwartz GK Munster PN. A phase I/II trial adding poly(ADP-ribose) polymerase (PARP) inhibitor veliparib to induction carboplatin-paclitaxel (Carbo-Tax) in patients with head and neck squamous cell carcinoma (HNSCC) Alliance A091101. J Clin Oncol. (2018) 36:6031–1. doi: 10.1200/jco.2018.36.15_suppl.6031

[71] Rodler ET Gralow J Kurland BF Griffin M Yeh R Thompson JA. Phase I: Veliparib with cisplatin (CP) and vinorelbine (VNR) in advanced triple-negative breast cancer (TNBC) and/or BRCA mutation-associated breast cancer. J Clin Oncol. (2014) 32:2569–9. doi: 10.1200/jco.2014.32.15_suppl.2569

[72] Thaker PH Salani R Brady WE Lankes HA Cohn DE Mutch DG. A phase I trial of paclitaxel, cisplatin, and veliparib in the treatment of persistent or recurrent carcinoma of the cervix: an NRG Oncology Study (NCT01281852). Ann Oncol. (2017) 28:505–11. doi: 10.1093/annonc/mdw635 PMC5815561 27998970

[73] Tsang ES Dhawan MS Pacaud R Thomas S Grabowsky J Wilch L. Synthetic lethality beyond BRCA: A phase I study of rucaparib and irinotecan in metastatic solid tumors with homologous recombination-deficiency mutations beyond BRCA1/2. JCO Precis Oncol. (2024) 8:e2300494. doi: 10.1200/po.23.00494 38865673

[74] Gottardo NG Endersby R Billups C Orr B Hansford JR Hassall T. MDB-65. Results from the sj-eliot phase 1 clinical trial evaluating prexasertib (ly2606368) in combination with cyclophosphamide or gemcitabine for children and adolescents with refractory or recurrent medulloblastoma. Neuro-Oncol. (2024) 26:0–0. doi: 10.1093/neuonc/noae064.514

[75] Moore KN Chambers SK Hamilton EP Chen L Oza AM Ghamande SA. Adavosertib with chemotherapy in patients with primary platinum-resistant ovarian, fallopian tube, or peritoneal cancer: an open-label, four-arm, phase II study. Clin Cancer Res. (2021) 28:158. doi: 10.1158/1078-0432.ccr-21-0158 34645648

[76] Gonzalez-Ochoa E Milosevic M Corr B Abbruzzese JL Girda E Miller RW. A phase I study of the Wee1 kinase inhibitor adavosertib (AZD1775) in combination with chemoradiation in cervical, upper vaginal, and uterine cancers. Int J Gynecol Cancer. (2023) 33:1208–14. doi: 10.1136/ijgc-2023-004491 PMC10711936 37380217

[77] Burris HA Berlin J Arkenau T Cote GM Lolkema MP Ferrer-Playan J. A phase I study of ATR inhibitor gartisertib (M4344) as a single agent and in combination with carboplatin in patients with advanced solid tumours. Br J Cancer. (2024) 130:1131–40. doi: 10.1038/s41416-023-02436-2 PMC10991509 38287179

[78] Keenan TE Li T Vallius T Guerriero JL Tayob N Kochupurakkal B. Clinical efficacy and molecular response correlates of the WEE1 inhibitor adavosertib combined with cisplatin in patients with metastatic triple-negative breast cancer. Clin Cancer Res. (2021) 27:983–91. doi: 10.1158/1078-0432.ccr-20-3089 PMC7887044 33257427

[79] Mittra A Coyne GHO Do KT Piha-Paul SA Kummar S Takebe N. Safety and tolerability of veliparib, an oral PARP inhibitor, and M6620 (VX-970), an ATR inhibitor, in combination with cisplatin in patients with refractory solid tumors. J Clin Oncol. (2019) 37:3067–7. doi: 10.1200/jco.2019.37.15_suppl.3067

[80] Ohnuma T Holland JF Goel S Wilck E Lehrer D Ghalib MH. Final results of a phase I dose-escalation study of ON 01910.Na in combination with oxaliplatin in patients with advanced solid tumors. J Clin Oncol. (2011) 29:e13584. doi: 10.1200/jco.2011.29.15_suppl.e13584

[81] Shapiro GI Wesolowski R Devoe C Lord S Pollard J Hendriks BS. Phase 1 study of the ATR inhibitor berzosertib in combination with cisplatin in patients with advanced solid tumours. Br J Cancer. (2021) 125:520–7. doi: 10.1038/s41416-021-01406-w PMC8367944 34040174

[82] Goff LW Azad NS Stein S Whisenant JG Koyama T Vaishampayan U. Phase I study combining the aurora kinase a inhibitor alisertib with mFOLFOX in gastrointestinal cancer. Invest N Drugs. (2019) 37:315–22. doi: 10.1007/s10637-018-0663-0 PMC6401337 30191522

[83] Dubois SG Mosse YP Fox E Kudgus RA Reid JM McGovern R. Phase 2 trial of alisertib in combination with irinotecan and temozolomide for patients with relapsed or refractory neuroblastoma. Clin Cancer Res. (2018) 24:1381. doi: 10.1158/1078-0432.ccr-18-1381 PMC6295246 30093449

[84] Pellini B Li J Schell MJ Melendez M Tanvetyanon T Creelan BC. A phase II trial of AZD1775 plus carboplatin-paclitaxel in squamous cell lung cancer (SqCLC). J Clin Oncol. (2024) 42:8545–5. doi: 10.1200/jco.2024.42.16_suppl.8545

[85] Wehler T Thomas M Schumann C Bosch-Barrera J Segarra NV Dickgreber NJ. A randomized, phase 2 evaluation of the CHK1 inhibitor, LY2603618, administered in combination with pemetrexed and cisplatin in patients with advanced nonsquamous non-small cell lung cancer. Lung Cancer. (2017) 108:212–6. doi: 10.1016/j.lungcan.2017.03.001 28625637

[86] Javed SR Lord S Badri SE Harman R Holmes J Kamzi F. CHARIOT: a phase I study of berzosertib with chemoradiotherapy in oesophageal and other solid cancers using time to event continual reassessment method. Br J Cancer. (2024) 130:467–75. doi: 10.1038/s41416-023-02542-1 PMC10844302 38129525

[87] Ahn DH Barzi A Ridinger M Samuëlsz E Subramanian RA Croucher PJP. Onvansertib in combination with FOLFIRI and bevacizumab in second-line treatment of KRAS-mutant metastatic colorectal cancer: A phase ib clinical study. Clin Cancer Res. (2024) 30:OF1–9. doi: 10.1158/1078-0432.ccr-23-3053 PMC11094418 38231047

[88] Conroy R. Onvansertib Yields Positive Activity in SCLC and Pancreatic Cancer (2023). Available online at: https://www.cancernetwork.com/view/onvansertib-yields-positive-activity-in-sclc-and-pancreatic-cancer (Accessed January 23, 2025).

[89] Kato H de Souza P Kim S-W Lickliter JD Naito Y Park K. Safety, pharmacokinetics, and clinical activity of adavosertib in combination with chemotherapy in Asian patients with advanced solid tumors: phase ib study. Target Oncol. (2020) 15:75–84. doi: 10.1007/s11523-020-00701-5 32034630 PMC7028795

[90] Oza AM Estevez-Diz M Grischke E-M Hall M Marmé F Provencher D. A biomarker-enriched, randomized phase II trial of adavosertib (AZD1775) plus paclitaxel and carboplatin for women with platinum-sensitive TP53-mutant ovarian cancer. Clin Cancer Res. (2020) 26:4767–76. doi: 10.1158/1078-0432.ccr-20-0219 32611648

[91] Jones R Plummer R Moreno V Carter L Roda D Garralda E. A phase I/II trial of oral SRA737 (a chk1 inhibitor) given in combination with low-dose gemcitabine in patients with advanced cancer. Clin Cancer Res. (2022) 29:331–40. doi: 10.1158/1078-0432.ccr-22-2074 PMC10539020 36378548

[92] Slotkin EK Mauguen A Ortiz MV Cruz FSD O’Donohue T Kinnaman MD. A phase I/II study of prexasertib in combination with irinotecan in patients with relapsed/refractory desmoplastic small round cell tumor and rhabdomyosarcoma. J Clin Oncol. (2022) 40:11503–3. doi: 10.1200/jco.2022.40.16_suppl.11503

[93] Study Details | Berzosertib + Topotecan in Relapsed Platinum-Resistant Small-Cell Lung Cancer (DDRiver SCLC 250). ClinicalTrials.gov. Available at: https://clinicaltrials.gov/study/NCT04768296?cond=NCT04768296. 10.1016/j.esmoop.2025.105918 PMC12794503 41421295

[94] Merck Advances Development Programs in Oncology Focusing on Novel Mechanisms and Pathways | Business Wire (2022). Available online at: https://www.businesswire.com/news/home/20220602005775/en/Merck-Advances-Development-Programs-in-Oncology-Focusing-on-Novel-Mechanisms-and-Pathways (Accessed January 23, 2025).

[95] Takahashi N Hao Z Villaruz LC Zhang J Ruiz J Petty WJ. Berzosertib plus topotecan vs topotecan alone in patients with relapsed small cell lung cancer. JAMA Oncol. (2023) 9:1669–77. doi: 10.1001/jamaoncol.2023.4025 PMC10570917 37824137

[96] Markham A. Pamiparib: first approval. Drugs. (2021) 81:1343–8. doi: 10.1007/s40265-021-01552-8 34287805

[97] Meador CB Digumarthy S Yeap BY Farago AF Heist RS Marcoux JP. Phase I/II investigator-initiated study of olaparib and temozolomide in SCLC: Updated analysis and CNS outcomes. J Clin Oncol. (2022) 40:8565–5. doi: 10.1200/jco.2022.40.16_suppl.8565 39470668

[98] Ingham M Allred JB Chen L Das B Kochupurakkal B Gano K. Phase II study of olaparib and temozolomide for advanced uterine leiomyosarcoma (NCI protocol 10250). J Clin Oncol. (2023) 41:4154–63. doi: 10.1200/jco.23.00402 PMC10852403 37467452

[99] Gabrielson A Tesfaye AA Marshall JL Pishvaian MJ Smaglo B Jha R. Phase II study of temozolomide and veliparib combination therapy for sorafenib-refractory advanced hepatocellular carcinoma. Cancer Chemother Pharmacol. (2015) 76:1073–9. doi: 10.1007/s00280-015-2852-2 PMC4612326 26449224

[100] Su JM Thompson P Adesina A Li X-N Kilburn L Onar-Thomas A. A phase I trial of veliparib (ABT-888) and temozolomide in children with recurrent CNS tumors: a Pediatric Brain Tumor Consortium report†. Neuro-Oncol. (2014) 16:1661–8. doi: 10.1093/neuonc/nou103 PMC4232081 24908656

[101] Middleton MR Friedlander P Hamid O Daud A Plummer R Falotico N. Randomized phase II study evaluating veliparib (ABT-888) with temozolomide in patients with metastatic melanoma. Ann Oncol. (2015) 26:2173–9. doi: 10.1093/annonc/mdv308 26202595

[102] Grignani G D’Ambrosio L Pignochino Y Palmerini E Zucchetti M Boccone P. Trabectedin and olaparib in patients with advanced and non-resectable bone and soft-tissue sarcomas (TOMAS): an open-label, phase 1b study from the Italian Sarcoma Group. Lancet Oncol. (2018) 19:1360–71. doi: 10.1016/s1470-2045(18)30438-8 30217671

[103] Merlini A Centomo ML Ferrero G Chiabotto G Miglio U Berrino E. DNA damage response and repair genes in advanced bone and soft tissue sarcomas: An 8-gene signature as a candidate predictive biomarker of response to trabectedin and olaparib combination. Front Oncol. (2022) 12:844250. doi: 10.3389/fonc.2022.844250 36110934 PMC9469659

[104] Perez J Soto M Quevedo C Alonso C Cepeda V Fuertes M. Poly(ADP-ribose) polymerase-1 inhibitor 3-aminobenzamide enhances apoptosis induction by platinum complexes in cisplatin-resistant tumor cells. Med Chem. (2006) 2:47–53. doi: 10.2174/157340606775197697 16787355

[105] Matulonis UA Monk BJ. PARP inhibitor and chemotherapy combination trials for the treatment of advanced Malignancies: does a development pathway forward exist? Ann Oncol. (2017) 28:443–7. doi: 10.1093/annonc/mdw697 28057663

[106] Zhou H Liu Q Zhang D Li Q Cao D Cheng N. Efficacy and safety of an oral combination therapy of niraparib and etoposide in platinum resistant/refractory ovarian cancer: a single arm, prospective, phase II study. Int J Gynecol Cancer. (2024) 34:1761–7. doi: 10.1136/ijgc-2024-005386 39074931

[107] Ryu M-H Kim H-D Oh D-Y Lee K-W Rha SY Kim ST. Association between homologous recombination deficiency (HRD) gene mutations and the efficacy of venadaparib in combination with irinotecan as third- or fourth-line treatment in patients with metastatic gastric cancer (mGC). J Clin Oncol. (2024) 42:e16057. doi: 10.1200/jco.2024.42.16_suppl.e16057

[108] Samol J Ranson M Scott E Macpherson E Carmichael J Thomas A. Safety and tolerability of the poly(ADP-ribose) polymerase (PARP) inhibitor, olaparib (AZD2281) in combination with topotecan for the treatment of patients with advanced solid tumors: a phase I study. Invest N Drugs. (2012) 30:1493–500. doi: 10.1007/s10637-011-9682-9 21590367

[109] Kummar S Chen A Ji J Zhang Y Reid JM Ames M. Phase I study of PARP inhibitor ABT-888 in combination with topotecan in adults with refractory solid tumors and lymphomas. Cancer Res. (2011) 71:5626–34. doi: 10.1158/0008-5472.can-11-1227 PMC3166628 21795476

[110] Takahashi N Desai PA Sciuto L Nichols S Steinberg SM Thomas A. Targeting genomic instability in extrapulmonary small cell neuroendocrine cancers: A phase II study with ATR inhibitor berzosertib and topotecan. J Clin Oncol. (2022) 40:8518–8. doi: 10.1200/jco.2022.40.16_suppl.8518

[111] Konstantinopoulos PA Cheng S-C Hendrickson AEW Penson RT Schumer ST Doyle LA. Berzosertib plus gemcitabine versus gemcitabine alone in platinum-resistant high-grade serous ovarian cancer: a multicentre, open-label, randomised, phase 2 trial. Lancet Oncol. (2020) 21:957–68. doi: 10.1016/s1470-2045(20)30180-7 PMC8023719 32553118

[112] Plummer R Dean E Arkenau H-T Redfern C Spira AI Melear JM. A phase 1b study evaluating the safety and preliminary efficacy of berzosertib in combination with gemcitabine in patients with advanced non-small cell lung cancer. Lung Cancer. (2022) 163:19–26. doi: 10.1016/j.lungcan.2021.11.011 34894455

[113] Middleton MR Dean E Evans TRJ Shapiro GI Pollard J Hendriks BS. Phase 1 study of the ATR inhibitor berzosertib (formerly M6620, VX-970) combined with gemcitabine ± cisplatin in patients with advanced solid tumours. Br J Cancer. (2021) 125:510–9. doi: 10.1038/s41416-021-01405-x PMC8368196 34040175

[114] Leijen S van Geel RMJM Pavlick AC Tibes R Rosen L Razak ARA. Phase I study evaluating WEE1 inhibitor AZD1775 as monotherapy and in combination with gemcitabine, cisplatin, or carboplatin in patients with advanced solid tumors. J Clin Oncol. (2016) 34:4371–80. doi: 10.1200/jco.2016.67.5991 PMC7845944 27601554

[115] Leijen S van Geel RMJM Sonke GS de Jong D Rosenberg EH Marchetti S. Phase II study of WEE1 inhibitor AZD1775 plus carboplatin in patients with TP53-mutated ovarian cancer refractory or resistant to first-line therapy within 3 months. J Clin Oncol. (2016) 34:4354–61. doi: 10.1200/jco.2016.67.5942 27998224

[116] Montagnoli A Rainoldi S Ciavolella A Ballinari D Caprera F Ceriani L. Abstract 1223: NMS-P293, a novel potent and selective PARP-1 inhibitor with high antitumor efficacy and tolerability. Cancer Res. (2016) 76:1223–3. doi: 10.1158/1538-7445.am2016-1223

[117] Illuzzi G Staniszewska AD Gill SJ Pike A McWilliams L Critchlow SE. Preclinical characterization of AZD5305, a next generation, highly selective PARP1 inhibitor and trapper. Clin Cancer Res. (2022) 28:4724–36. doi: 10.1158/1078-0432.ccr-22-0301 PMC9623235 35929986

[118] O’Brien MER Wigler N Inbar M Rosso R Grischke E Santoro A. Reduced cardiotoxicity and comparable efficacy in a phase IIItrial of pegylated liposomal doxorubicin HCl(CAELYXTM/Doxil^®^) versus conventional doxorubicin forfirst-line treatment of metastatic breast cancer. Ann Oncol. (2004) 15:440–9. doi: 10.1093/annonc/mdh097 14998846

[119] Barenholz Y. Doxil^®^ — The first FDA-approved nano-drug: Lessons learned. J Control Release. (2012) 160:117–34. doi: 10.1016/j.jconrel.2012.03.020 22484195

[120] Perez-Fidalgo JA Tavira B Peña CJ Guerra E Martínez-Pretel JJ García Y. Role of the receptor for advanced glycation end products (RAGE) in blood as a potential biomarker for progression to olaparib: A *post hoc* analysis of patients with platinum-resistant ovarian cancer (PROC) treated in the ROLANDO-GEICO 1601 trial. J Clin Oncol. (2024) 42:5566–6. doi: 10.1200/jco.2024.42.16_suppl.5566

[121] Perez-Fidalgo JA Cortés A Guerra E García Y Iglesias M Sarmiento UB. Olaparib in combination with pegylated liposomal doxorubicin for platinum-resistant ovarian cancer regardless of BRCA status: a GEICO phase II trial (ROLANDO study) ☆. ESMO Open. (2021) 6:100212. doi: 10.1016/j.esmoop.2021.100212 34329939 PMC8446804

[122] Conte GD Sessa C von Moos R Viganò L Digena T Locatelli A. Phase I study of olaparib in combination with liposomal doxorubicin in patients with advanced solid tumours. Br J Cancer. (2014) 111:651–9. doi: 10.1038/bjc.2014.345 PMC4134498 25025963

[123] Ohmoto A Yachida S. Current status of poly(ADP-ribose) polymerase inhibitors and future directions. OncoTargets Ther. (2017) 10:5195–208. doi: 10.2147/ott.s139336 PMC5667784 29138572

[124] Zhang H Kreis J Schelhorn S-E Dahmen H Grombacher T Zühlsdorf M. Mapping combinatorial drug effects to DNA damage response kinase inhibitors. Nat Commun. (2023) 14:8310. doi: 10.1038/s41467-023-44108-y 38097586 PMC10721915

